# A ventilator strategy combining low tidal volume ventilation, recruitment maneuvers, and high positive end-expiratory pressure does not increase sedative, opioid, or neuromuscular blocker use in adults with acute respiratory distress syndrome and may improve patient comfort

**DOI:** 10.1186/s13613-014-0033-9

**Published:** 2014-11-06

**Authors:** Sangeeta Mehta, Deborah J Cook, Yoanna Skrobik, John Muscedere, Claudio M Martin, Thomas E Stewart, Lisa D Burry, Qi Zhou, Maureen Meade

**Affiliations:** 1 Department of Medicine and Interdepartmental Division of Critical Care Medicine, Mount Sinai Hospital, University of Toronto, 600 University Avenue, Suite 18-216, Toronto M5G 1X5, Ontario, Canada; 2 Interdepartmental Division of Critical Care, Hamilton Health Sciences and Departments of Medicine and Clinical Epidemiology and Biostatistics, McMaster University, Hamilton L8S 4K1, Ontario, Canada; 3 Département de Medicine, Soins Intensifs, Hôpital Maisonneuve Rosemont, Universite de Montreal, Montreal H1T 2M4, Quebec, Canada; 4 Department of Medicine, Kingston General Hospital, Queen’s University, Kingston K7L 2V7, Ontario, Canada; 5 Department of Medicine, London Health Sciences Centre, Western University, London N6A 5A5, Ontario, Canada; 6 Niagara Health System, St. Catharines L2S 0A9, Ontario, Canada; 7 Department of Pharmacy and Medicine, Mount Sinai Hospital, Toronto M5G 1X5, Ontario, Canada; 8 Department of Clinical Epidemiology and Biostatistics, McMaster University, Hamilton L8S 4K1, Ontario, Canada

**Keywords:** ARDS, Neuromuscular blocker, Sedation, Opioid, Mechanical ventilation, Clinician comfort

## Abstract

**Background:**

The Lung Open Ventilation Study (LOV Study) compared a low tidal volume strategy with an experimental strategy combining low tidal volume, lung recruitment maneuvers, and higher plateau and positive end-expiratory pressures (PEEP) in adults with acute respiratory distress syndrome (ARDS). Herein, we compared sedative, opioid, and neuromuscular blocker (NMB) use among patients managed with the intervention and control strategies and clinicians' assessment of comfort in both groups.

**Methods:**

This was an observational substudy of the LOV Study, a randomized trial conducted in 30 intensive care units in Canada, Australia, and Saudi Arabia. In 16 centers, we recorded daily doses of sedatives, opioids, and NMBs and surveyed bedside clinicians about their own comfort with the assigned ventilator strategy and their perceptions of patient comfort. We compared characteristics and outcomes of patients who did and did not receive NMBs.

**Results:**

Study groups received similar sedative, opioid, and NMB dosing on days 1, 3, and 7. Patient comfort as assessed by clinicians was not different in the two groups: 93% perceived patients had no/minimal discomfort. In addition, 92% of clinicians were comfortable with the assigned ventilation strategy without significant differences between the two groups. When clinicians expressed discomfort, more expressed discomfort about PEEP levels in the intervention vs control group (2.9% vs 0.7%, *P* <0.0001), and more perceived patient discomfort among controls (6.0% vs 4.3%, *P* = 0.049). On multivariable analysis, the strongest associations with NMB use were higher plateau pressure (hazard ratio (HR) 1.15; 95% confidence interval (CI) 1.07 to 1.23; *P* = 0.0002) and higher daily sedative dose (HR 1.03; 95% CI 1.02 to 1.05; *P* <0.0001). Patients receiving NMBs had more barotrauma, longer durations of mechanical ventilation and hospital stay, and higher mortality.

**Conclusions:**

In the LOV Study, high PEEP, low tidal volume ventilation did not increase sedative, opioid, or NMB doses in adults with ARDS, compared with a lower PEEP strategy, and appeared at least as comfortable for patients. NMB use may reflect worse lung injury, as these patients had more barotrauma, longer durations of ventilation, and higher mortality.

**Trial registration:**

ClinicalTrials.gov Identifier NCT00182195

## Background

Low tidal volume ventilation is a standard of care in adults with the acute respiratory distress syndrome (ARDS) and is associated with reduced mortality compared to traditional tidal volumes [1]. Low tidal volume ventilation may reduce minute ventilation with resultant patient discomfort and patient-ventilator asynchrony, and greater use of sedation, opioids, and neuromuscular blockers (NMBs). However, post hoc analyses of patients enrolled in two participating centers of the original low tidal volume trial did not detect differences in the dose or duration of sedatives and opioids between low tidal volume and control groups [2],[3].

Despite concerns about NMBs including the need for deep sedation and the risk of ICU-acquired weakness [4], results of a recent trial have renewed interest in the use of NMBs in patients with ARDS. Papazian and colleagues randomized 340 patients with early severe ARDS to receive continuous infusions of cisatracurium besylate or placebo for 48 h and observed a 30% relative reduction in adjusted mortality risk in the cisatracurium-treated group, as well as more ventilator-free days and no increase in the risk of acquired paresis in the intensive care unit (ICU) [5].

The Lung Open Ventilation Study (LOV Study) was a multicenter randomized trial that compared a standard low tidal volume ventilation strategy with a novel strategy designed to recruit the lung, combining low tidal volumes, high positive end-expiratory pressure (PEEP), and recruitment maneuvers in 983 adults with ARDS [6]. The original publication reported no differences in the proportion of patients receiving sedative, opioid, and NMB infusions, or the duration of these infusions, but did not provide detailed data about medication administration, including specific medications and dosages. Given the benefits of sedation minimization [7] and the potential benefit of NMBs [5], it is important to better understand the use of these medications in patients managed with high PEEP, low tidal volume, and recruitment maneuvers - a combination commonly used in patients with ARDS.

In this substudy of the LOV Study, our objectives were to 1) describe the types and doses of sedatives, opioids, and NMBs administered to patients mechanically ventilated using a high PEEP, low tidal volume and recruitment maneuver strategy (intervention group) versus conventional low tidal volume ventilation (control group); 2) describe clinicians' assessment of comfort in patients randomized to the two groups; and 3) compare characteristics and outcomes of patients who did and did not receive NMBs. We hypothesized that clinicians would have more concerns about discomfort in the intervention group, resulting in the use of higher doses of sedatives and opioids, because they were managed with recruitment maneuvers and significantly higher plateau pressures, PEEP, and I/E ratio [6].

## Methods

### Study design

The complete methods and results of the LOV Study have been previously published [6]. This substudy of the LOV Study was planned and initiated after 539 of 983 patients had been enrolled in the primary study. For this substudy, sedative, opioid, and NMB data were collected in 16 centers (13 in Canada, 2 in Australia, 1 in Saudi Arabia), on 444 subsequent patients, between August 2000 and March 2006. These data were collected retrospectively for patients enrolled between August 2000 and January 2003, and prospectively for patients enrolled after January 2003. Clinician comfort questionnaires were completed for 417 patients. The research ethics board of each hospital approved the trial (see Additional file 1 for ethics boards), and *a priori* informed consent was obtained from substitute decision makers.

### Participants

The LOV Study included 983 patients ≥18 years of age with acute respiratory distress syndrome, defined as new respiratory symptoms within 28 days with bilateral radiographic opacities, and a ratio of arterial oxygen tension to inspired oxygen fraction (PaO_2_/FIO_2_) ≤250. We excluded patients with left atrial hypertension as the cause of respiratory failure, expected duration of mechanical ventilation <48 h, >48 h of eligibility, inability to wean from nitric oxide, severe chronic respiratory or neuromuscular disease, intracranial hypertension, morbid obesity (>1 kg/cm body height), pregnancy, lack of commitment to life support, >50% anticipated 6-month mortality, and participation in a confounding trial. Use of sedatives, opioids, and NMBs was at the discretion of the ICU clinicians.

### Data collection and outcome measurements

Research personnel recorded demographic characteristics, physiological data, and relevant laboratory data from the 24 h preceding randomization. At baseline and daily, we documented doses and route of administration of all sedatives, opioids, NMBs, and three anti-psychotics (haloperidol, risperidone, and olanzapine). At each center, we documented whether sedation, analgesia, or NMBs were administered using a local protocol.

For each of the first four study days, we administered an anonymous, patient-specific questionnaire to physicians and respiratory therapists, surveying their assessment of patient comfort, manifestations of the patient's discomfort, the clinician's own level of comfort with the ventilator strategy, and reasons for their discomfort, where appropriate (please see Additional file 1 for the questionnaire). We collected these data for 417 consecutive patients enrolled in the LOV Study after 13 June 2001.

### Statistical analysis

We present data regarding doses of medications administered on study days 1, 3, and 7 for 444 patients. Continuous data are reported as mean and standard deviation (SD), or median and interquartile range (IQR) when not normally distributed. Categorical variables are reported as frequency and percentage. We compared medication doses and durations, and clinicians' assessment of patient comfort in patients randomized to the intervention and control groups. We also compared baseline characteristics and outcomes of all 983 patients enrolled in the LOV Study who received and did not receive NMBs. For the comparisons of continuous data, we used Student's *t* test if it was normally distributed or Wilcoxon's rank-sum test if it was not normally distributed. For categorical data, we used the chi-square test for the comparison, or Fisher's exact test when the expected event count was less than 5. All statistical tests were two-tailed, and a *P* value <0.05 was considered statistically significant.

To obtain hazard ratios (HRs) for time to NMB use, we performed Cox regression analysis and used the marginal model approach to account for variation due to clustering by center. The independent variables included baseline factors (age in 10-year increments, Acute Physiology and Chronic Health Evaluation II (APACHE II) score in 5-point increments, sepsis, and ventilator strategy) and time-dependent repeated measurements which reflected the most recent values at each point in time (sedative daily doses in 10 mg midazolam equivalent increments, opioid daily doses in 10 mg morphine equivalent increments, levels of PEEP and *P*_plateau_, PaO_2_/FiO_2_ in 5-unit increments, tidal volume, and barotrauma). To calculate midazolam equivalents, the following conversion was used: 0.5 mg lorazepam = 1 mg midazolam; to calculate morphine equivalents, the following conversion was used: 10 mg morphine = 100 mcg fentanyl = 2 mg hydromorphone [8]. Hazard ratios are presented with 95% confidence intervals. Risk factors that had a strong association (*P* <0.01) were considered significant.

## Results

### Study population

Table 1 shows baseline characteristics of the 444 patients in this substudy. Among these patients, there were no differences in baseline characteristics of patients randomized to the intervention and control groups, nor were there differences between patients in this substudy and the entire LOV Study population.

**Table 1:** Baseline characteristics

**Characteristics**	**Intervention group**	**Control group**
	***N*** **=218**	***N*** **=226**
Age, mean (SD)	53.5 (16.2)	55.5 (16.6)
Female sex, *n* (%)	91 (41.7)	90 (39.8)
APACHE II score, mean (SD)	25.6 (7.9)	26.4 (7.8)
PaO_2_/FiO_2_, mean (SD)	145.5 (43.5)	149.2 (51.6)
Set PEEP, cm H_2_O, mean (SD)	11.2 (3.4)	10.9 (3.2)
*P*_plateau_, cm H_2_O, mean (SD)	30.4 (5.6)	30.1 (6.1)
PaCO_2_, mean (SD)	43.0 (11.2)	42.1 (9.5)
pH, mean (SD)	7.4 (0.1)	7.4 (0.1)
Tidal volume, ml/kg PBW, mean (SD)	8.4 (2.1)	8.2 (2.0)
Minute ventilation, mean (SD)	11.9 (3.6)	11.9 (3.9)
Glasgow Coma Score, mean (SD)	6.2 (3.9)	6.4 (4.0)
Hepatic failure/cirrhosis, *n* (%)	11 (5.1)	8 (3.5)
Chronic dialysis, *n* (%)	22 (10.1)	21 (9.3)
Corticosteroids, *n* (%)	52 (51.5)	49 (48.5)
Sedative infusions, *n* (%)	166 (76.2)	178 (78.8)
Opioid infusions, *n* (%)	150 (68.8)	162 (71.7)
Neuromuscular blockers, *n* (%)	42 (19.3)	39 (17.3)

In this table, we present baseline characteristics of all included patients. Data are presented as *n* (%) or mean and standard deviation. APACHE II, Acute Physiology and Chronic Health Evaluation II. Sedative infusions = benzodiazepines and/or propofol.

### Sedative, opioid, and antipsychotic administration

On days 1, 3, and 7, approximately 91%, 87%, and 78% of patients received intravenous sedation, with similar proportions in the two groups (Additional file 1: Table S1). Midazolam was the most common sedative (61% to 78% of patients on days 1, 3, and 7), followed by propofol (20% to 28%) and lorazepam (9% to 16%); more than 75% of patients had midazolam and propofol administered as continuous infusions. Fewer than 2% of patients had sedation managed using a protocol. There were no differences between groups in median daily sedative doses on days 1, 3, and 7 (Table 2).

**Table 2:** Intravenous sedative and opioid administration on days 1, 3, and 7

**Variables**	**Day 1**			**Day 3**			**Day 7**		
	**Intervention**	**Control**	***P*** **value**	**Intervention**	**Control**	***P*** **value**	**Intervention**	**Control**	***P*** **value**
	***N*** **=218**	***N*** **=226**		***N*** **=203**	***N*** **=219**		***N*** **=144**	***N*** **=163**	
Any sedation^a^, *n* (%)	199 (91.3)	208 (92.0)	0.96	178 (87.7)	192 (87.7)	0.99	115 (79.9)	127 (77.9)	0.68
Midazolam									
mg, median (IQR)	92 (32 to 194)	81 (36 to 157)	0.30	118 (46 to 240)	101 (34 to 240)	0.20	107 (24 to 240)	193 (52.5 to 352)	0.06
*n*	172	178		145	162		94	100	
Lorazepam									
mg, median (IQR)	7 (3 to 19)	12 (4 to 35)	0.31	5 (2 to 12)	8 (2 to 32)	0.49	9 (3 to 24)	9 (2 to 48)	0.90
*n*	23	32		19	35		20	26	
Propofol									
mg, median (IQR)	1,040 (200 to 2,470)	860 (260 to 2,160)	0.48	1,300 (280 to 2,830)	1,340 (285 to 2,840)	0.69	1,180 (270 to 3,125)	1,758 (470 to 3,250)	0.98
*n*	50	49		55	44		41	33	
Any opioid^b^, *n* (%)	182 (83.5)	193 (85.4)	0.74	161 (79.3)	180 (82.2)	0.45	104 (72.2)	120 (73.6)	0.78
Morphine									
mg, median (IQR)	72 (124 to 176)	89 (25 to 178)	0.83	96 (40 to 240)	80 (32 to 212)	0.97	95 (24.5 to 237.5)	94 (26.5 to 249)	0.30
*n*	134	131		118	121		84	80	
Fentanyl									
mcg, median (IQR)	1,600 (690 to 3,225)	1,270 (550 to 3,100)	0.47	2,495 (495 to 4,350)	2,728 (760 to 4,800)	0.40	3,072 (1,335 to 4,100)	2,240 (580 to 5,200)	0.51
*n*	60	58		46	58		20	40	
Hydromorphone									
mg, median (IQR)	4 (4 to 4)	72 (8 to 97)	0.44	34 (2 to 65)	98 (40 to 136)	0.44	5 (5 to 5)	69 (49 to 88)	0.60
*n*	1	3		2	3		1	2	

In this table, we present the percentage of patients receiving any sedation, any opioid, and median daily doses of sedatives and opioids in the two groups, on days 1, 3, and 7. The total *N* for each group at each timepoint represents the number of patients for whom data was reported. Doses are presented as median and interquartile range (IQR). *n* represents the number of patients receiving the sedative/opioid agent on the corresponding day. ^a^Sedation includes any of the following agents: lorazepam, midazolam, propofol, ketamine, clonazepam, and diazepam. ^b^Opioids include morphine, meperidine, fentanyl, sufentanil, alfentanil, and codeine.

On days 1, 3, and 7, more than 70% of patients received intravenous opioids, with the greatest frequency of use on day 1 (Additional file 1: Table S2). Morphine was the most common opioid (49% to 60% patients), followed by fentanyl (14% to 27%) and hydromorphone (0.5% to 1.4%). Morphine and fentanyl were administered as continuous infusions to more than 75% and 90% of patients, respectively. Fewer than 1% of patients had opioids managed using a protocol. The overall proportion of patients receiving opioids was similar in both groups. There were no significant differences in median daily opioid doses between groups on days 1, 3, and 7 (Table 2).

On days 1, 3, and 7, fewer than 10% of patients received an antipsychotic medication (haloperidol, risperidone, or olanzapine), with similar proportions of patients and doses in the intervention and control groups (Additional file 1: Table S3).

### Clinician comfort assessment

Clinicians completed 2,740 questionnaires for 417 patients, during the first four study days (Table 3). Clinicians assessed patient comfort in both groups as similar. The majority (>93%) perceived that patients had no or minimal discomfort. When asked about manifestations of patient discomfort, the most common response was ventilator asynchrony for both groups. Patients in the control group were more often perceived to have ventilator asynchrony (10% vs 8%, *P* = 0.02) and diaphoresis (1% vs 0.3%, *P* = 0.03). Clinicians indicated on 92.2% of surveys that they were ‘not at all’ or ‘minimally’ uncomfortable with the assigned ventilator strategy; clinician comfort was similar between the two ventilation strategies. Of clinicians who expressed any discomfort about the assigned strategy, the two most common concerns in both groups were the need for large amounts of sedation or paralysis (8.7% vs 6.1%, *P* = 0.01) and apparent patient discomfort (6.0% vs 4.3%, *P* = 0.049); these concerns were greater in control group patients. More clinicians expressed discomfort about high levels of PEEP in the intervention than the control group (2.9% vs 0.7%, *P* <0.0001).

**Table 3:** Comfort assessment: perceptions of physicians and respiratory therapists

	**Intervention**	**Control**	**Relative risk (95% confidence interval)**	***P*** **value**
	***N*** **=1,297**	***N*** **=1,443**		
How much discomfort is this patient experiencing? *n* (%)	*N* =1,185	*N* =1,309		
No discomfort	960 (81.0)	1,020 (77.9)	1.04 (0.99, 1.08)	0.06
Minimal discomfort	152 (12.8)	192 (14.7)	0.87 (0.72, 1.07)	0.18
Moderate, major, or extreme discomfort	73 (6.2)	97 (7.4)	0.83 (0.62, 1.11)	0.22
How does the patient's discomfort manifest? *n* (%)	*N* =1,229	*N* =1,360		
Air hunger	40 (3.3)	55 (4.0)	0.80 (0.54, 1.20)	0.29
Agitation	92 (7.5)	122 (9.0)	0.83 (0.64, 1.08)	0.17
Ventilator asynchrony	97 (7.9)	143 (10.5)	0.75 (0.59, 0.96)	0.02
Diaphoresis	4 (0.3)	14 (1.0)	0.32 (0.10, 0.96)	0.03
How uncomfortable are you with the ventilator strategy? *n* (%)	*N* =1,186	*N* =1,308		
Not at all	1,002 (84.5)	1,082 (82.7)	1.02 (0.99, 1.06)	0.24
Minimally	105 (8.9)	136 (10.4)	0.85 (0.67, 1.08)	0.19
Moderately	56 (4.7)	62 (4.7)	0.996 (0.70, 1.42)	0.98
Very	17 (1.4)	19 (1.5)	0.99 (0.52, 1.89)	0.97
Extremely	6 (0.5)	9 (0.7)	0.74 (0.26, 2.06)	0.56
What is the cause of your discomfort? *n* (%)	*N* =1,223	*N* =1,357		
High level of PEEP	35 (2.9)	10 (0.7)	3.88 (1.93, 7.81)	<0.0001
Apparent patient discomfort	52 (4.3)	81 (6.0)	0.71 (0.51, 1.00)	0.049
Need for large amounts of sedation or paralysis	75 (6.1)	118 (8.7)	0.71 (0.53, 0.93)	0.01
Concerns of other bedside clinician	15 (1.2)	17 (1.3)	0.98 (0.49, 1.95)	0.95
Concerns of family	5 (0.4)	13 (1.0)	0.43 (0.15, 1.19)	0.10

In this table, we present results of the comfort assessments. Daily for the first 4 days after randomization, we administered an anonymous questionnaire to physicians and respiratory therapists, asking about their assessment of the patient's discomfort related to the assigned ventilation strategy, the manifestation of the patient's discomfort, the clinician's discomfort with the ventilator strategy, and the reasons for the clinician's discomfort (see Additional file 1 for the questionnaire). This table represents 417 patients and 2,740 questionnaires; 2,322 (85%) of questionnaires were completed by physicians and 418 (15%) by respiratory therapists. The median number of questionnaires per patient was 4 (IQR 4 to 8).

### Neuromuscular blocker administration

On days 1, 3, and 7, approximately 26%, 20%, and 13% of the 444 substudy patients received NMBs, with similar proportions in the two groups (Additional file 1: Table S4). Fewer than 2% of patients had NMBs managed using a protocol. The most commonly used NMB was vecuronium, followed by cisatracurium, rocuronium, and pancuronium; median daily doses of the three most commonly used agents were similar in the two groups on days 1, 3 and 7 (data not shown).

Table 4 shows baseline characteristics and outcomes of all 983 patients enrolled in the LOV Study who received and did not receive NMBs. Overall, 431 (43.8%) patients received NMBs, and they were started on day 1 (median, IQR 1 to 4 days). For these patients, the median number of days receiving a NMB was 3 (IQR 1 to 5 days); both the intervention and control groups received NMBs for a similar number of days. At baseline, patients who received NMBs were younger and had a higher severity of illness, with higher APACHE II score, lower pH and PaO_2_/FiO_2_, higher PEEP, higher plateau pressure, higher PaCO_2_, and lower tidal volume. In Cox regression analysis of the 444 substudy patients (Table 5), higher daily sedative dose (HR 1.03; 95% confidence interval (CI) 1.02 to 1.05; *P* <0.0001) and higher plateau pressure (HR 1.15; 95% CI 1.07 to 1.23; *P* =0.0002) were independently associated with time to NMB use. Regarding outcomes, patients who received NMBs had more barotrauma, longer durations of mechanical ventilation and hospital stay, and higher mortality.

**Table 4:** Baseline characteristics and outcomes of patients who received and did not receive neuromuscular blockers

**Variables**	**NMB**	**No NMB**	***P*** **value**
	***n*** **= 431**	***n*** **= 552**	
Baseline characteristics			
Age, years, mean (SD)	54.4 (16.7)	56.9 (16.3)	0.02
Female sex, *n* (%)	165 (38.3)	229 (41.5)	0.31
APACHE II score, mean (SD)	26.7 (7.3)	24.3 (7.9)	<0.0001
PaO_2_/FiO_2_, mean (SD)	132.0 (48.5)	154.6 (46.3)	<0.0001
Set PEEP, cm H_2_O, mean (SD)	12.0 (3.5)	10.7 (3.1)	<0.0001
*P*_plateau_, cm H_2_O, mean (SD)	30.9 (6.0)	28.7 (5.4)	<0.0001
PaCO_2_, mean (SD)	44.2 (11.1)	40.7 (8.4)	<0.0001
pH, mean (SD)	7.35 (0.08)	7.38 (0.08)	<0.0001
Tidal volume, ml/kg PBW, mean (SD)	8.2 (2.2)	8.6 (2.1)	0.01
Minute ventilation, l/min, mean (SD)	11.6 (3.5)	11.4 (3.4)	0.20
Barotrauma, *n* (%)	19 (4.4)	17 (3.1)	0.27
Hepatic failure/cirrhosis, *n* (%)	18 (4.2)	26 (4.7)	0.69
Chronic dialysis, *n* (%)	34 (7.9)	62 (11.2)	0.08
Interventions and outcomes			
Corticosteroids during first 28 days, *n* (%)	227 (52.7)	183 (33.2)	<0.0001
Mechanical ventilation, days, median (IQR)	14 (8 to 23)	8 (5 to 12)	<0.0001
Hospital stay, days, median (IQR)	24 (14 to 44)	20 (11 to 37)	0.01
Death during first 28 days, *n* (%)	168 (37.0)	131 (23.7)	<0.0001
Death in ICU, *n* (%)	191 (44.3)	132 (23.9)	<0.0001
Death during mechanical ventilation, * n* (%)	185 (43.5)	119 (22.1)	<0.0001
Barotrauma^a^, *n* (%)	93 (21.6)	69 (12.5)	0.0001

In this table, we compare baseline characteristics and outcomes in all 983 patients enrolled in the LOV Study according to whether they received neuromuscular blockers (NMBs) or not. SD, standard deviation; APACHE II, Acute Physiology and Chronic Health Evaluation II; MOD, multiple organ dysfunction; PEEP, positive end-expiratory pressure; PBW, predicted body weight; ICU, intensive care unit. ^a^Regardless of baseline status of barotrauma; 34 of 93 patients developed barotrauma after NMB administration.

**Table 5:** Cox's proportional-hazards regression analysis - time to neuromuscular blocker use as the outcome and stratified by center

	**Adjusted HR (95% CI)**	***P*** **value**
Baseline variables		
Age, 10-year increase	1.01 (0.87, 1.17)	0.92
APACHE II score, 5-point increase	1.66 (1.04, 2.66)	0.04
Intervention versus control	1.17 (0.32, 4.30)	0.82
Sepsis	1.00 (0.51, 1.97)	0.99
Time-dependent variables		
Sedative doses, 10-mg increase	1.03 (1.02, 1.05)	<0.0001
Opioid doses, 10-mg increase	1.01 (0.999, 1.02)	0.07
PEEP	0.88 (0.70, 1.11)	0.28
*P*_plateau_	1.15 (1.07, 1.23)	0.0002
PaO_2_/FiO_2_, 5-point increase	1.05 (1.03, 1.08)	0.03
Tidal volume (ml/kg PBW)	0.99 (0.67, 1.47)	0.95
Barotrauma	8.54 (1.17, 62.09)	0.03

In this table, we present the multivariable Cox regression results. Both baseline and time-dependent variables were considered. APACHE II, Acute Physiology and Chronic Health Evaluation II. Sedative doses were expressed in midazolam equivalents, and opioid doses were expressed in fentanyl equivalents.

## Discussion

In this substudy of the LOV Study, we found that the addition of high PEEP and recruitment maneuvers to a low tidal volume ventilation strategy did not alter the overall dosing or duration of sedatives, opioids, or NMBs, compared with a low tidal volume strategy, despite higher plateau pressures. While clinicians were generally comfortable with both ventilator strategies, our results suggest that clinicians more frequently perceived patient discomfort in the control group, with more ventilator asynchrony and, rarely, diaphoresis. In addition, they were more frequently concerned about high amounts of sedation and paralysis among control patients, contrary to our hypothesis. Our assessment of NMB use among all patients in the LOV Study showed that patients who received NMBs were younger and had more severe ARDS and worse outcomes, including more barotrauma, longer durations of mechanical ventilation and hospital stay, and higher mortality.

While low tidal volume ventilation is a standard of care in the management of adult ARDS, the role for higher versus lower PEEP strategies is less clear. Three multicenter trials assessing unique high-PEEP protocols did not observe clear benefit or harm with respect to patient survival [6],[9],[10]. However, a subsequent individual patient data meta-analysis of these trials found a statistically significant reduction in mortality with higher PEEP among the subgroup of patients with ARDS, rather than milder forms of acute lung injury [11]. These findings suggest that a higher PEEP strategy is likely as effective as a lower PEEP strategy and perhaps superior for patients with more severe disease. Our findings in this substudy of the LOV Study, suggesting that bedside clinicians perceive more patient discomfort with a lower PEEP strategy, provide additional support for clinicians who perceive their patients will benefit from a high-PEEP ventilation strategy in ARDS. Overall, clinicians were equally comfortable with the two ventilator strategies and administered similar doses of sedatives and analgesics to patients in the two groups.

Our results are also similar to a retrospective analysis of the multicenter ALVEOLI trial, which randomized 549 patients with acute lung injury/ARDS to a high PEEP or a lower PEEP strategy, and found no difference in survival between the two groups [9],[12]. In the ALVEOLI trial, the compared ventilation strategies differed primarily with respect to PEEP levels. In contrast, the two LOV Study groups differed significantly with respect to more respiratory variables: PEEP levels, plateau pressures, I/E ratio, and the use of recruitment maneuvers. In the ALVEOLI trial, sedatives and opioids were used in more than 80% of patients, in a similar proportion in both groups; however, a higher proportion of patients in the lower PEEP group received NMBs (45% vs 33%, *P* = 0.006) and for a longer duration (1.1 vs 0.9 days, *P* = 0.008). The use of sedatives and opioids, but not NMBs, was associated with longer time on mechanical ventilation.

Over the last few decades, clinicians have avoided NMBs in critically ill patients because of concerns regarding the need for deep sedation, as well as the risk of ICU-acquired weakness [4]. However, a recent trial in patients with severe ARDS demonstrated potential benefits [5]. In this multicenter, double blind trial, 340 patients with early severe ARDS were randomized to receive cisatracurium besylate or placebo for 48 h. After adjusting for the pre-specified covariates of baseline PaO_2_/FiO_2_, Simplified Acute Physiology II score, and plateau pressure, in patients with a PaO_2_/FiO_2_ of less than 120, the mortality rate was 30.8% in the cisatracurium group compared with 44.6% in the control group (HR 0.68; 95% confidence interval 0.48 to 0.98; *P* = 0.04) - a 30% relative mortality reduction. Additionally, the cisatracurium group had more ventilator-free days and no increased incidence of ICU-acquired weakness. Two systematic reviews of 431 patients in three randomized trials reported lower mortality, more ventilator-free days, and less barotrauma in patients treated with NMBs [13],[14].

This recent trial by Papazian and colleagues [5] prompted us to further evaluate NMB use in the LOV Study. We found that patients who received NMBs were younger, had a higher APACHE II score, more barotrauma, longer durations of mechanical ventilation and hospital stay, and higher mortality. Their worse outcomes likely reflect greater illness severity, and it is not possible to draw inferences about causality with respect to the use of NMBs. In our multivariable analysis, we identified higher plateau pressure and higher sedative doses as independent determinants of NMB use, which both likely reflect more severe ARDS.

To the best of our knowledge, this is the only study to date which has evaluated clinician comfort and clinicians' assessment of patient discomfort with the assigned ventilator strategies. Strengths of this study are the daily prospective recording of sedative, analgesic, and NMB administration in relation to carefully protocolized ventilation strategies, and real-time assessment of perceived patient comfort associated with these exposures during the conduct of a trial. The sample size is large compared with previous studies, and we conducted multivariable regression analysis to address confounding associations with NMBs. The multicenter design enhances the generalizability of these findings. This study has several limitations. The administration of sedatives, opioids, and NMBs was managed by unblinded ICU clinicians rather than protocolized, and we have no information about the use of sedation or pain scales, delirium, NMB monitoring, or ICU-acquired weakness in this study. The more frequent clinician perception of ventilator asynchrony in the control group may relate to the use of volume-assist control mode, compared with pressure control mode in the intervention group [15].

## Conclusions

Patients with ARDS are commonly managed with a ventilator strategy that includes low tidal volume, high PEEP, and recruitment maneuvers. In the LOV Study, the use of such a strategy was not associated with more sedative, opioid, or neuromuscular blocker use, nor with higher doses, compared with low tidal volume ventilation. Overall, bedside clinicians were equally comfortable with these two ventilator strategies though they perceived more patient discomfort with the lower PEEP strategy. Patients who received NMBs had longer lengths of stay and higher mortality, likely reflecting their greater severity of illness.

## Abbreviations

ARDS: acute respiratory distress syndrome

HR: hazard ratio

ICU: intensive care unit

IQR: interquartile range

LOV Study: Lung Open Ventilation Study

NMB: neuromuscular blocker

PEEP: positive end-expiratory pressure

SD: standard deviation

## Competing interests

The authors declare that they have no competing interests.

## Authors’ contributions

SM, DJC, YS, JM, CMM, TES, LDB, and MM conceived the study, and participated in its design. SM drafted the manuscript. QZ participated in the design of the study and performed the statistical analysis. All authors critically revised the manuscript for important intellectual content, and approved the final manuscript.

## Additional file

## Supplementary Material

Additional file 1: **Supporting information.** A file showing the research ethics boards of institutions that approved the study, the 4-day comfort assessment form, and four supplementary tables. Click here for file
