# Supplementary material for: A ventilator strategy combining low tidal volume ventilation, recruitment maneuvers, and high positive end-expiratory pressure does not increase sedative, opioid, or neuromuscular blocker use in adults with acute respiratory distress syndrome and may improve patient comfort
Source: Ann Intensive Care. 2014 Nov 6;4:33. doi: 10.1186/s13613-014-0033-9 (PMC4273695; doi:10.1186/s13613-014-0033-9)
Supplement: Additional file 1: — Supporting information. A file showing the research ethics boards of institutions that approved the study, the 4-day comfort assessment form, and four supplementary tables. [file s13613-014-0033-9-S1.doc]

Additional File

A ventilator strategy combining low tidal volume ventilation, recruitment maneuvers and high positive end-expiratory pressure does not increase sedative, opioid, or neuromuscular blocker use in adults with Acute Respiratory Distress Syndrome, and may improve patient comfort

Sangeeta Mehta, Deborah J Cook, Yoanna Skrobik, John Muscedere, Claudio M Martin, Thomas E Stewart, Lisa D Burry, Qi Zhou, Maureen Meade for the Lung Open Ventilation Study Investigators

Page

Research ethics boards 2

4-day Comfort Assessment Form 3

Table 1. Intravenous sedation administration on days 1, 3 and 7. 4

Table 2. Intravenous opioid administration on days 1, 3 and 7. 5

Table 3. Antipsychotic medication administration on days 1, 3 and 7. 6

Table 4. Use of neuromuscular blockers on days 1, 3 and 7. 7

The research ethics boards of the following institutions approved the study.

Canada:

Centre Hospitalier Universitaire de Sherbrook, Sherbrook, Quebec

Charles LeMoyne Hospital, Montreal, Quebec

Hamilton Health Science, General Hospital, Hamilton, Ontario

Hamilton Health Sciences, Henderson Hospital, Hamilton, Ontario

Hamilton Health Sciences, McMaster, Hamilton, Ontario

Hopital de l’Enfant Jésus, Québec City, Québec

Hopital Maisonneuve Rosemont, Montreal, Quebec

Hotel Dieu Grace, Windsor, Ontario

Jewish General Hospital, Montreal, Quebec

London Health Sciences Centre, University Hospital, London, Ontario

London Health Sciences Centre, Victoria Hospital, London, Ontario

Montreal General Hospital, Montreal, Quebec

Mount Sinai Hospital, Toronto, Ontario

Ottawa Hospital, Civic Campus, Ottawa, Ontario

Ottawa Hospital, General Campus, Ottawa, Ontario

Royal Columbian Hospital, New Westminster, British Columbia

Royal Victoria Hospital, Montreal, Quebec

St. Joseph’s Healthcare, Hamilton, Ontario

St. Michael’s Hospital, Toronto, Ontario

St. Paul’s Hospital, Vancouver, British Columbia

Sunnybrook Hospital, Toronto, Ontario

Toronto General Hospital, Toronto, Ontario

Toronto Western Hospital, Toronto, Ontario

University of Alberta Hospital, Edmonton, Alberta

Vancouver General Hospital, Vancouver, British Columbia

Vancouver Island Health Research Centre, Victoria, British Columbia

Australia:

Alfred Hospital, Melbourne

Royal Prince Alfred Hospital, Camperdown

Western Hospital, Victoria

Saudi Arabia:

Medical City King Fahad National Guard Hospital, Riyadh

Additional File Table 1. Intravenous sedation administration on days 1, 3 and 7: proportion of patients receiving bolus therapy, continuous infusions, or both.

| Variables | Day 1 | | | Day 3 | | | Day 7 | | |
| --- | --- | --- | --- | --- | --- | --- | --- | --- | --- |
|  | Intervention  N=218 | Control  N=226 | P | Intervention  N=203 | Control  N=219 | P | Intervention  N=144 | Control  N=163 | P |
| Any Sedation^1^, n (%) | 199 (91.3) | 208 (92.0) | 0.96 | 178 (87.7) | 192 (87.7) | 0.99 | 115 (79.9) | 127 (77.9) | 0.68 |
| Midazolam, n (%)  Bolus only  Infusion only  Infusion + bolus | 172 (78.9)  28 (12.8)  106 (48.6)  38 (17.4) | 178 (78.7)  16 (7.1)  120 (53.1)  42 (18.6) | 0.82  0.04  0.40  0.79 | 145 (71.4)  24 (11.8)  87 (42.9)  34 (16.8) | 162 (74.0)  20 (9.1)  108 (49.3)  34 (15.5) | 0.56  0.37  0.18  0.73 | 94 (65.3)  21 (14.6)  59 (41.0)  14 (9.7) | 100 (61.4)  10 (6.1)  68 (41.7)  22 (13.5) | 0.48  0.01  0.89  0.31 |
| Lorazepam, n (%)  Bolus only  Infusion only  Infusion + bolus | 23 (10.6)  19 (8.7)  1 (0.5)  3 (1.4) | 32 (14.2)  23 (10.2)  5 (2.2)  1 (0.4) | 0.26  0.62  0.22  0.36 | 19 (9.4)  15 (7.4)  2 (1.0)  1 (0.5) | 35 (16.0)  29 (13.2)  3 (1.4)  0 | 0.04  0.049  1  0.48 | 20 (13.9)  15 (10.4)  2 (1.4)  1 (0.7) | 26 (16.0)  20 (12.3)  4 (2.5)  0 | 0.61  0.69  0.47  1 |
| Propofol, n (%)  Bolus only  Infusion only  Infusion + bolus | 50 (22.9)  8 (3.7)  34 (15.6)  8 (3.7) | 50 (22.1)  8 (3.5)  38 (16.8)  4 (1.8) | 0.80  0.93  0.76  0.25 | 55 (27.1)  7 (3.5)  43 (21.2)  5 (2.5) | 44 (20.1)  6 (2.7)  34 (15.5)  4 (1.8) | 0.09  0.67  0.13  0.74 | 41 (28.5)  3 (2.1)  34 (23.6)  4 (2.8) | 33 (20.3)  4 (2.5)  23 (14.1)  6 (3.7) | 0.09  1  0.03  0.75 |

Legend for Table 1. In this table we present the numbers and percentages of patients in the two groups who received any sedation, specific sedation agents, and the use of only intermittent boluses or continuous infusions, on days 1, 3 and 7.

1. Sedation is defined as any of the following agents: lorazepam, midazolam, propofol, ketamine, clonazepam, diazepam. Patients could receive more than one sedative.

Additional File Table 2. Intravenous opioid administration on days 1, 3 and 7: proportion of patients receiving bolus therapy, continuous infusions, or both.

| Variables | Day 1 | | | Day 3 | | | Day 7 | | |
| --- | --- | --- | --- | --- | --- | --- | --- | --- | --- |
|  | Intervention  N=218 | Control  N=226 | P | Intervention  N=203 | Control  N=219 | P | Intervention  N=144 | Control  N=163 | P |
| Any opioid^1^, n (%) | 182 (83.5) | 193 (85.4) | 0.74 | 161 (79.3) | 180 (82.2) | 0.45 | 104 (72.2) | 120 (73.6) | 0.78 |
| Morphine, n (%)  Bolus only  Infusion only  Infusion + bolus | 131 (60.0)  24 (11.0)  70 (32.1)  37 (17.0) | 134 (59.3)  27 (11.9)  75 (33.2)  32 (14.2) | 0.77  0.78  0.86  0.39 | 118 (58.1)  17 (8.4)  72 (35.5)  29 (14.3) | 121 (55.3)  21 (9.6)  67 (30.6)  32 (14.6) | 0.55  0.66  0.29  0.92 | 84 (58.3)  21 (14.6)  48 (33.3)  15 (10.4) | 80 (49.1)  14 (8.6)  51 (31.3)  15 (9.2) | 0.10  0.10  0.70  0.72 |
| Fentanyl, n (%)  Bolus only  Infusion only  Infusion + bolus | 58 (26.6)  4 (1.8)  44 (20.2)  10 (4.6) | 61 (27.0)  5 (2.2)  50 (22.1)  6 (2.7) | 0.97  1  0.65  0.27 | 46 (22.7)  5 (2.5)  35 (17.2)  6 (3.0) | 58 (26.5)  3 (1.4)  48 (21.9)  7 (3.2) | 0.36  0.49  0.23  0.89 | 20 (13.9)  1 (0.7)  16 (11.1)  3 (2.1) | 40 (24.5)  2 (1.2)  31 (19.0)  6 (3.7) | 0.02  1  0.05  0.51 |
| Hydromorphone, n (%)  Bolus only  Infusion only  Infusion + bolus | 1 (0.5)  1 (0.5)  0  0 | 3 (1.3)  1 (0.4)  1 (0.4)  1 (0.4) | 0.62  1  1  1 | 2 (1.0)  1 (0.5)  1 (0.5)  0 | 3 (1.4)  0  2 (0.9)  1 (0.5) | 1  0.48  1  1 | 1 (0.7)  0  1 (0.7)  0 | 2 (1.2)  0  1 (0.6)  1 (0.6) | 1  /  1  1 |

Legend for Table 2. In this table we present the numbers and percentages of patients in the two groups who received any intravenous opioid, specific opioids administered, and the use of only intermittent boluses or continuous infusions, on days 1, 3 and 7.

1. Opioids include morphine, meperidine, fentanyl, sufentanil, alfentanil, and codeine. Patients could receive more than one opioid.

Additional File Table 3. Antipsychotic medication administration on days 1, 3 and 7

| Variables | Day 1 | | Day 3 | | Day 7 | |
| --- | --- | --- | --- | --- | --- | --- |
|  | Intervention  N=218 | Control  N=226 | Intervention  N=203 | Control  N=219 | Intervention  N=144 | Control  N=163 |
| Haloperidol (mg)  n (%) | 10 (2-20)  5 (2.2) | 8 (3-20)  7 (3.1) | 15 (9.5-20)  16 (7.9) | 22.5 (20-35)  10 (4.6) | 17.5 (5-25)  6 (4.2) | 20 (10-20)  9 (5.5) |
| Risperidone (mg)  n (%) | /  0 | /  0 | /  0 | 1 (1-1)  1 (0.5%) | 5 (5-5)  1 (0.7%) | /  0 |
| Olanzepine (mg)  n (%) | 5 (5-5)  1 (0.5) | 5 (3-10)  3 (1.3) | 5 (5-15)  3 (1.5) | 4 (3-7.5)  4 (1.8) | 5 (5-5)  2 (1.4) | 3 (3-3)  2 (1.2) |

Legend for Table 3. In this table we present the numbers and percentages of substudy patients in the two groups who received haloperidol, risperidone, or olanzepine, and the median doses of each antipsychotic medication. Risperidone and olanzapine were administered enterally. Of the patients who received haloperidol, most received it intravenously; enteral haloperidol was administered to 1 patient in the control group on day 1, and to 1 patient in each group on day 3.

Doses are presented as median (interquartile range). There were no significant differences in antipsychotic use between intervention and control groups.

Additional File Table 4. Use of neuromuscular blockers on days 1, 3 and 7.

| Variables | Day 1 | | | Day 3 | | | Day 7 | | |
| --- | --- | --- | --- | --- | --- | --- | --- | --- | --- |
|  | Intervention  N=218 | Control  N=226 | P | Intervention  N=204 | Control  N=219 | P | Intervention  N=144 | Control  N=163 | P |
| Any NMB^a^, n (%) | 60 (27.5) | 59 (26.1) | 0.69 | 39 (19.1) | 47 (21.5) | 0.55 | 16 (11.1) | 26 (16.0) | 0.22 |
| Vecuronium, n (%)  Bolus only  Infusion only  Infusion + bolus | 20 (9.2)  9 (4.1)  6 (2.8)  5 (2.3) | 22 (9.7)  10 (4.4)  5 (2.2)  7 (3.1) | 0.87  0.89  0.70  0.61 | 14 (6.9)  5 (2.5)  7 (3.4)  2 (1.0) | 18 (8.2)  6 (2.7)  10 (4.6)  2 (0.9) | 0.60  0.85  0.55  1 | 4 (2.8)  2 (1.4)  2 (1.4)  0 | 10 (6.1)  3 (1.8)  6 (3.7)  1 (0.6) | 0.18  1  0.29  1 |
| Cisatracurium, n (%)  Bolus only  Infusion only  Infusion + bolus | 19 (8.7)  5 (2.3)  11 (5.0)  3 (1.4) | 18 (8)  4 (1.8)  12 (5.3)  2 (0.9) | 0.75  0.75  0.92  0.68 | 14 (6.9)  0  14 (6.9)  0 | 13 (5.9)  2 (0.9)  8 (3.7)  3 (1.4) | 0.70  0.50  0.14  0.25 | 10 (6.9)  1 (0.7)  7 (4.9)  2 (1.4) | 8 (4.9)  2 (1.2)  4 (2.5)  2 (1.2) | 0.45  1  0.36  1 |
| Rocuronium, n (%)  Bolus only  Infusion only  Infusion + bolus | 18 (8.3)  17 (7.8)  1 (0.5)  0 | 15 (6.6)  13 (5.8)  2 (0.9)  0 | 0.50  0.38  1  / | 6 (2.9)  4 (2.0)  0  2 (1.0) | 12 (5.5)  11 (5.0)  1 (0.5)  0 | 0.20  0.12  1  0.23 | 1 (0.7)  0  0  1 (0.7) | 6 (3.7)  2 (1.2)  2 (1.2)  2 (1.2) | 0.13  0.50  0.50  1 |
| Pancuronium, n (%)  Bolus only  Infusion only  Infusion + bolus | 3 (1.4)  3 (1.4)  0  0 | 5 (2.2)  3 (1.3)  1 (0.4)  1 (0.4) | 0.72  1  1  1 | 4 (2.0)  4 (2.0)  0  0 | 5 (2.3)  3 (1.4)  2 (0.9)  0 | 1  0.72  0.50  / | 4 (2.8)  2 (1.4)  2 (1.4)  0 | 5 (3.1)  4 (2.5)  1 (0.6)  0 | 1  0.69  0.60  / |

Legend for Table 4. In this table we present the numbers and percentages of patients in the two groups who received any neuromuscular blocker (NMB), the specific agents administered, and the use of only intermittent boluses or continuous infusions, on days 1, 3 and 7.

LOV = Lung Open Ventilation Strategy.

^a^ Neuromuscular blockers include vecuronium, cisatracurium, pancuronium, rocuronium, and atracurium; atracurium was used in 1 patient in the intervention group.
